# Novel Radiographic Indexes for Elbow Stability Assessment: Part A—Cadaveric Validation

**DOI:** 10.1007/s43465-021-00407-4

**Published:** 2021-05-09

**Authors:** Francesco Luceri, Davide Cucchi, Enrico Rosagrata, Carlo Eugenio Zaolino, Marco Viganò, Laura de Girolamo, Andrea Zagarella, Michele Catapano, Mauro Battista Gallazzi, Paolo Angelo Arrigoni, Pietro Simone Randelli

**Affiliations:** 1 U.O.C. Clinica Ortopedica e Traumatologica Universitaria CTO, Azienda Socio Sanitaria Territoriale Centro Specialistico Ortopedico Traumatologico Gaetano Pini-CTO, Piazza Cardinal Ferrari 1, 20122 Milan, Italy; 2 Department of Orthopaedics and Trauma Surgery, Universitätsklinikum Bonn, Venurberg-Campus 1, 53127 Bonn, Germany; 3 Residency Program, Università Degli Studi di Milano, Via Mangiagalli 31, 20133 Milan, Italy; 4 Laboratorio di Biotecnologie Applicate All’Ortopedia, IRCCS Istituto Ortopedico Galeazzi, Milan, Italy; 5 Servizio di Radiologia, Azienda Socio Sanitaria Territoriale Centro Specialistico Ortopedico Traumatologico Gaetano Pini-CTO, Milan, Italy; 6 Laboratory of Applied Biomechanics, Department of Biomedical Sciences for Health, Università Degli Studi di Milano, Via Mangiagalli 31, 20133 Milan, Italy; 7 U.O.C. 1° Clinica Ortopedica, Azienda Socio Sanitaria Territoriale Centro Specialistico Ortopedico Traumatologico Gaetano Pini-CTO, Piazza Cardinal Ferrari 1, 20122 Milan, Italy; 8 Research Center for Adult and Pediatric Rheumatic Diseases (RECAP-RD), Department of Biomedical Sciences for Health, Università Degli Studi di Milano, Via Mangiagalli 31, 20133 Milan, Italy

**Keywords:** Elbow joint, Radiographic study, Cadaveric study, Ulna, Olecranon

## Abstract

**Introduction:**

Elbow bony stability relies primarily on the high anatomic congruency between the humeral trochlea and the ulnar greater sigmoid notch. No practical tools are available to distinguish different morphotypes of the proximal ulna and herewith predict elbow stability. The aim of this study was to assess inter-observer reproducibility, evaluate diagnostic performance and determine responsiveness to change after simulated coronoid process fracture for three novel elbow radiographic indexes.

**Methods:**

Ten fresh-frozen cadaver specimens of upper limbs from human donors were available for this study. Three primary indexes were defined, as well as two derived angles: Trochlear Depth Index (TDI); Posterior Coverage Index (PCI); Anterior Coverage Index (ACI); radiographic coverage angle (RCA); olecranon–diaphisary angle (ODA). Each index was first measured on standardized lateral radiographs and subsequently by direct measurement after open dissection. Finally, a type II coronoid fracture (Regan and Morrey classification) was created on each specimen and both radiographic and open measurements were repeated. All measurements were conducted by two orthopaedic surgeons and two dedicated musculoskeletal radiologists.

**Results:**

All three indexes showed good or moderate inter-observer reliability and moderate accuracy and precision when compared to the gold standard (open measurement). A significant change between the radiographic TDI and ACI before and after simulated coronoid fracture was observed [TDI: decrease from 0.45 ± 0.03 to 0.39 ± 0.08 (*p* = 0.035); ACI: decrease from 1.90 ± 0.17 to 1.58 ± 0.21 (*p* = 0.001)]. As expected, no significant changes were documented for the PCI. Based on these data, a predictive model was generated, able to identify coronoid fractures with a sensitivity of 80% and a specificity of 100%.

**Conclusion:**

New, simple and easily reproducible radiological indexes to describe the congruency of the greater sigmoid notch have been proposed. TDI and ACI change significantly after a simulated coronoid fracture, indicating a good responsiveness of these parameters to a pathological condition. Furthermore, combining TDI and ACI in a regression model equation allowed to identify simulated fractures with high sensitivity and specificity. The newly proposed indexes are, therefore, promising tools to improve diagnostic accuracy of coronoid fractures and show potential to enhance perioperative diagnostic also in cases of elbow instability and stiffness.

**Level of evidence:**

Basic science study.

**Clinical relevance:**

The newly proposed indexes are promising tools to improve diagnostic accuracy of coronoid fractures as well as to enhance perioperative diagnostic for elbow instability and stiffness.

## Introduction

Elbow stability is guaranteed by primary and secondary constraints [1–4]. The ulnohumeral articulation is the most important primary stabilizer of the elbow joint [5–7]. Bony stability relies primarily on the high anatomic congruency between the humeral trochlea and the ulnar greater sigmoid notch (GSN). This structure has a C-shaped concavity, extending between two bony processes, the olecranon and the coronoid [2, 8].

The coronoid process is the most important bony constraint against posterior elbow dislocation, together with the radial head [9–13]. With a loss of 50% or more of coronoid height, major translational, rotational and valgus–varus instability appears [14, 15].

The radiographic classification proposed by Regan and Morrey [16], inspired by this principle, aimed to define a simple diagnostic–therapeutic algorithm to approach coronoid fractures [17, 18].

Later on, O’Driscoll proposed a CT-based classification [19], which is anatomically more accurate, and is considered particularly useful for surgical planning and evaluation of complex fracture patterns [18].

Both classifications have strengths and limitations. Among the latter, the complete C-shaped olecranon morphology is not adequately examinated and, the anatomical congruency between the proximal ulna and the distal humerus is neglected, although it also plays a role in preventing elbow dislocation. Moreover, both classifications focus on the coronoid process only, giving no importance to the olecranon process as a possible factor affecting elbow joint stability.

The association between olecranon fractures and elbow dislocation or subluxation suggests a potential role of this anatomical structure in antero-posterior elbow stability [1, 20]. On the other hand, the olecranon is believed to play only a minor role in rotational and valgus–varus stability [21, 22].

Several scientific reports investigated the anatomical and biomechanical properties of the GSN and its contribution to elbow stability; however, without providing a practical application of this precious knowledge [23, 24].

Furthermore, simple, reliable and predictive parameters to describe the anatomical morphology of the proximal ulna are not available yet. The only accepted evidence is that the height of the coronoid process directly correlates with its resistance against posterior elbow dislocation [25, 26]. The creation of reliable, accurate radiographic parameters to describe GSN elbow coverage could provide clinicians with practical tools to determine the intrinsic osseous stability of the elbow and to describe different morphotypes of the proximal ulna, possibly predicting elbow stability.

The aim of this study was to assess the anatomical accuracy of three new elbow radiographic indexes that may be used to quantify the intrinsic osseous stability by comparing radiographic with open anatomical measurements on cadaveric specimens. The secondary aims of this study were to evaluate the inter-observer reproducibility of the indexes, their diagnostic performance and their responsiveness to change in case of a simulated coronoid fracture.

## Materials and methods

Ten fresh-frozen upper limbs cadaveric specimens from human donors including the complete middle third of the humerus and the entire hand were available for this study. Before investigation, care was taken to evaluate the specimens for visible signs of previous trauma, gross instability or deformity.

Plain radiographs in anteroposterior and lateral projections were then taken to visualize integrity of bony structures and joint congruency.

**Fig. 1:**
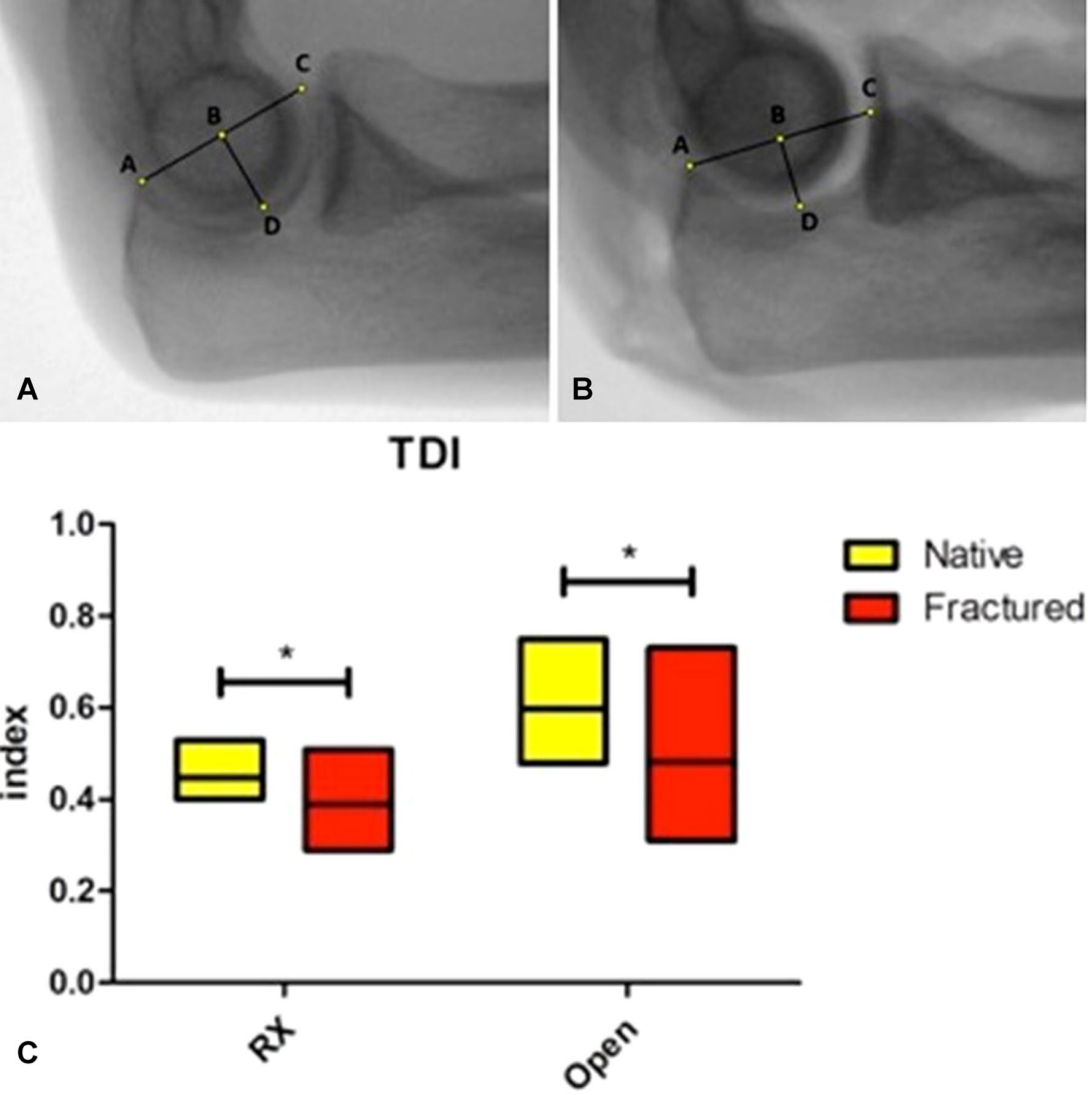
Digital elbow radiographs in lateral projection of the same elbow, depicted in its native state (**a**) and after simulated coronoid fracture (**b**). The Trochlear Depth Index (TDI) is the ratio between the distance from the olecranon to the coronoid tip (AC) and the distance between this line and the deepest point of the trochlea (TDI = BD/AC). Box plots (**c**) illustrating the comparison between the radiographic and open TDI in native elbows and after coronoid osteotomy (*p value < 0.05). Data represent minimum, maximum (box) and mean (line). *RX* Radiographic

**Fig. 2:**
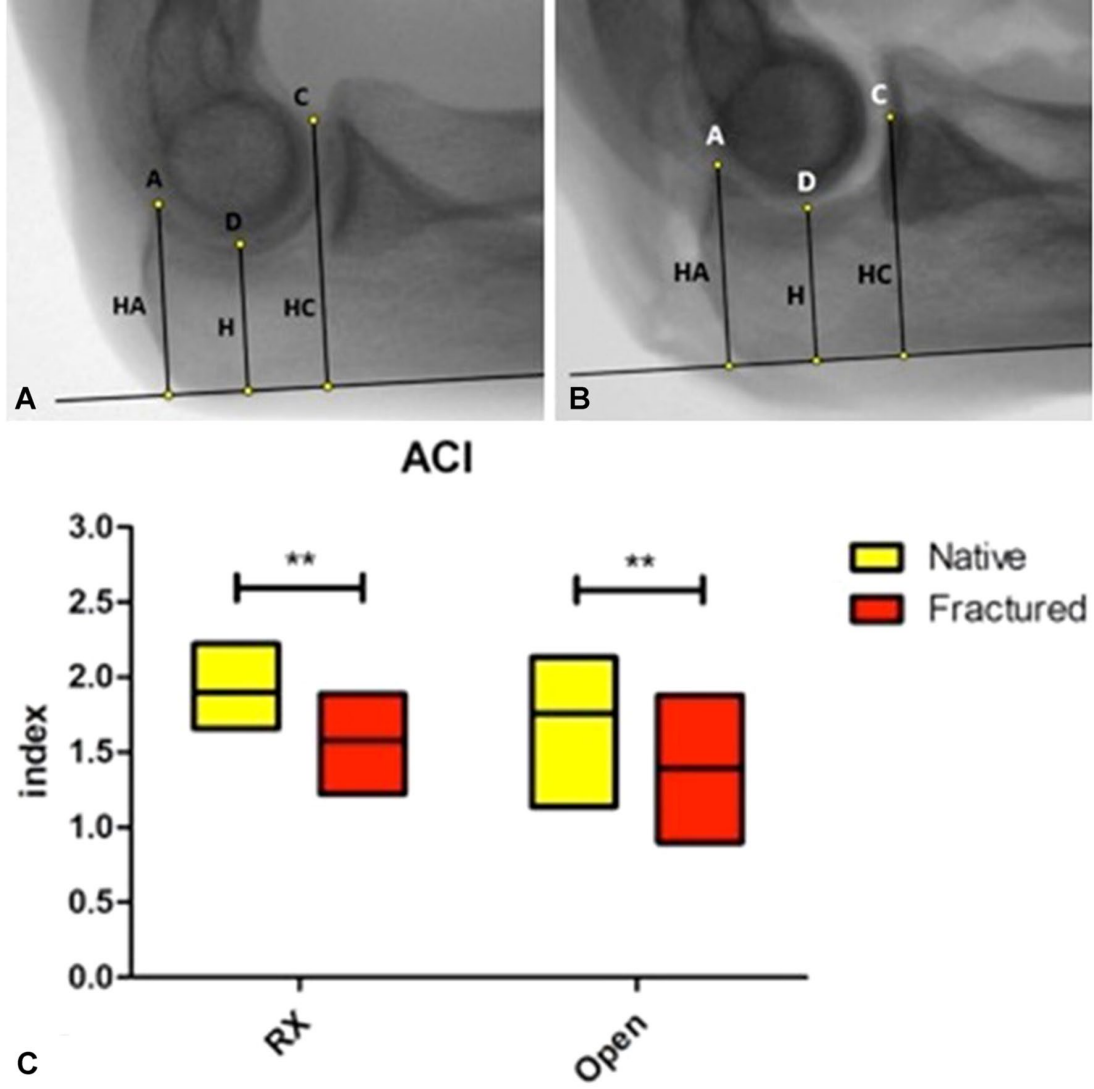
Digital elbow radiographs in lateral projection of the same elbow, depicted in its native state (**a**) and after simulated coronoid fracture (**b**). The Anterior Coverage Index (ACI) is the ratio between HC and H (ACI = HC/H); the Posterior Coverage Index (PCI) is the ratio between HA and H (PCI = HA/H). Box plots (**c**) illustrating the comparison between radiographic and open ACI in native and fractured elbows (**p value < 0.01). Data represent minimum, maximum (box) and mean (line). *RX* radiographic

To perform the *radiographic study* of the elbow, standardized medio-lateral digital radiographs were obtained holding the joint in 90° of flexion (Figs. 1A-B, 2A-B). The quality of the radiographic projection was considered appropriate when the contours of the throclear sulcus, of the capitellum and of the medial throclea were seen as three concentric circles or circular segments [27, 28].

**Fig. 3:**
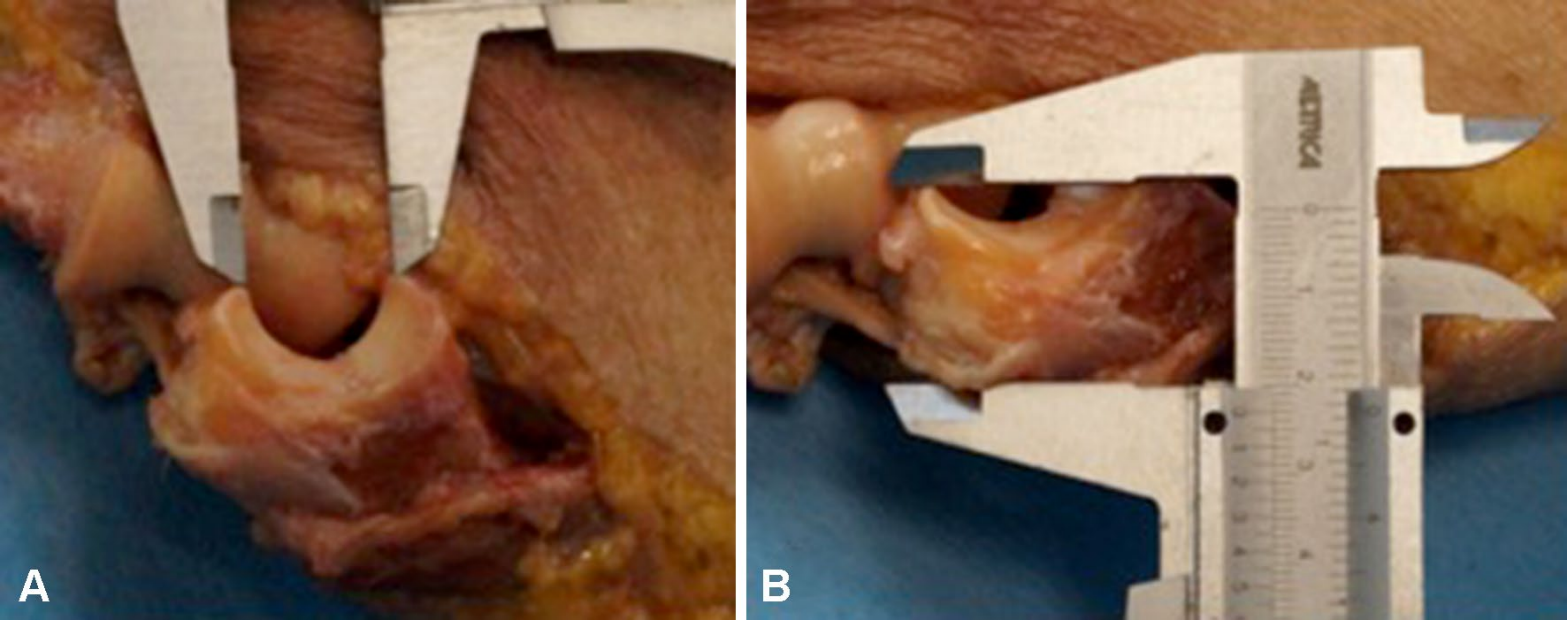
Open measurements; (**a**) olecranon–coronoid tip distance; (**b**) olecranon height

Subsequently, a medial approach centered on the medial epicondyle was performed on each specimen; the common flexor origin was identified, released from the humeral epicondyle and reflected distally. Similarly, the medial collateral ligament was identified and released, allowing dislocation of the ulno-humeral joint. The proximal ulna anatomical bony landmarks were finally identified by open dissection and linear distances (Olecranon–coronoid tip distance; olecranon height) were measured using a graduated sliding calliper (Fig. 3).

After measurement of normal anatomy on the intact specimen, a horizontal coronoid process osteotomy was performed with a chisel to simulate a Regan and Morrey type II coronoid fracture, since this type of lesion has been shown to entail a clinically significant loss of joint stability [16]. The osteotomy line was placed on a coronal plane at half of the distance between the coronoid tip and the bare area of the proximal ulna trochlear notch, as described in previous studies [29, 30]. After the osteotomy, a graduated sliding calliper was used to repeat the anatomical measurements relevant for the radiographic study. All linear measurements were performed by two observers reaching consensus on the obtained value by mutual agreement.

Finally, the fractured elbows were reduced manually, and a second medio-lateral digital radiograph was obtained for each specimen, with the same technical characteristics and quality criteria described above.

A single expert surgeon with extensive experience in elbow surgery performed all surgical procedures (P.A.). Institutional approval of the study protocol was obtained by the Nicola’s Foundation & ICLO Research Centre (ID 19506).

### Radiographic Study

All digital radiographs were exported as digital image files (.BMP) and analysed independently by four observers, not blinded to the performed procedure: two orthopaedic surgeons not involved in the surgical procedures (F.L., E.R.) and two dedicated musculoskeletal radiologists (A.Z., M.C.). The software GeoGebra Classic 5 Version 5.0.426.0 (GeoGebra GmbH, Altenbergerstraße 69, 4040 Linz, Austria) was used to mark radiographic landmarks and measure linear distances. Since all described parameters are ratios between linear measurements on the same radiograph, standardized scaling of the radiographic digital images was not necessary.

The following points and lines were identified on each radiograph:

- A: tip of the olecranon process;
- B: midpoint of the segment AC;
- C: tip of the coronoid process (or the most posterior point of the superior surface of the coronoid process in case of a simulated fracture);
- D: deepest point of the greater sigmoid notch (determined by the intersection of a line perpendicular to AC and passing through the point B and the greater sigmoid notch profile of the ulna);
- r: posterior olecranon cortex line.

The segments AC and BD were measured and the minimal trochlear height (HD, or simply H), the olecranon height (HA) and the coronoid height (HC) were determined as the linear distances between the points D, A, C and the posterior olecranon line.

Using these linear measurements, three primary indexes were developed:

1. **Trochlear Depth Index (TDI)**: defined as the ratio between proximal ulna trochlear notch depth (BD) and olecranon–coronoid distance (AC). *(TDI* = *BD/AC, where AC indicates the distance between coronoid and olecranon tips and BD indicates the shortest distance from AC to the deepest point of trochlear notch)* (Fig. 1).
2. **Posterior Coverage Index (PCI)**: defined as the ratio between the olecranon height (HA) and the minimal proximal ulna trochlear height (H). *(PCI* = *HA/H, where HA indicates the shortest distance between olecranon tip and the posterior olecranon cortex and H indicates the height of the deepest trochlear portion)* (Fig. 2).
3. **Anterior Coverage Index (ACI)**: defined as the ratio between the coronoid height (HC) and the minimal proximal ulna trochlear height (H). *(ACI* = *HC/H, where HC indicates shortest distance from coronoid tip to the posterior olecranon cortex and H indicates the height of the deepest trochlear portion)* (Fig. 2).

Furthermore, two derived angles were described:

1. **Radiographic Coverage Angle (RCA)**: defined as the dorsally opened angle subtended by the circular segment AC of the GSN. *RCA* = *4 ∙ arctan (2 ∙ BD/AC), where AC indicates the distance between coronoid and olecranon tips and BD indicates the shortest distance from AC to the deepest point of trochlear notch)*
2. **Olecranon–Diaphisary Angle (ODA)**: defined as the angle between the ulnar diaphysis and the line passing through AC. *ODA* = *arcsen [(HC-HA)/AC], where HC indicates shortest distance from coronoid tip to the posterior olecranon cortex, HA indicates the shortest distance between olecranon tip and the posterior olecranon cortex and AC indicates the distance between coronoid and olecranon tips)*

The three aforementioned primary indexes were measured on the radiographs, before and after coronoid osteotomy. Evaluation of the derived angles was outside the scope of the current study, as they can be derived by mathematical operations and they are not measurable ex vivo.

Finally, the above-described radiological parameters were calculated also from the linear measurements obtained after open cadaveric dissection. These measurements, defined as “open TDI”, “open ACI”, “open PCI” to distinguish them from their radiographic counterparts, were considered as gold standard for the subsequent statistical evaluation.

### Statistical Analysis

Statistical analyses were performed using R software (R Core Team, Wien, Austria). The Shapiro–Wilk test was used to evaluate data distribution. Interclass correlation was assessed using *icc* function from *irr* package. Accuracy for each parameter was reported as mean % error compared to the gold standard (open measurements with graduated calliper). For each sample, Δ accuracy was obtained as the difference between the mean of values reported by all observers and the value obtained by open measurement, normalized for the value of open measurement:

$$\Delta\, {\text{accuracy}} = \frac{{mean\, value\, in\,  RX-open \,measurement}}{open\, measurement}$$

The mean value from all samples was calculated to define the mean % error for each index.

Then, precision for each measurement was calculated as the ratio between the standard deviation (SD) and mean of the measurements provided by the four raters:

$$ {\text{precision}} = \frac{{standard\,deviation\, among \, observers}} {mean\, of \, measurement\, among\, observers} $$

The Student’s *t* test was used to assess differences between measurements in fractured and native samples for each parameter.

The mean measurements obtained by the four observers for each sample were used for the analysis.

Effect sizes were calculated as Cohen’s *d*, comparing indexes from native and fractured elbows:

$$ {d} = \frac{{native\,mean\, value-fractured \,mean \,value}} {pooled \,standard \,deviation}. $$

Cohen’s *d* values above 0.8 were considered as large effect sizes [31].

A generalized linear model was developed to evaluate the effect of TDI and ACI on the presence of fractures. The ACI effect was significant, and the addition of TDI improved the fitness of the model. Model fitting was measured by Akaike Information Criterion [32].

Sensitivity of the test was calculated as the ratio between true positives and the sum true positives + false negatives.

Specificity was calculated as the ratio between true negatives and the sum true negatives + false positives. Data were presented as mean ± SD. A *p* value < 0.05 was considered statistically significant.

A receiver operating characteristic (ROC) curve for the different threshold values was drawn and the area under curve was calculated [33] to evaluate the test performance.

## Results

Complete sets of radiographic and linear measurements were obtained for all ten cadaveric specimens (median age at death 59.3 years [47–69]; females: 50%, left elbow: 30%). No difficulties were encountered during the dissections and the measurements and two lateral digital radiographs of adequate quality were obtained for each specimen, before and after coronoid osteotomy.

### Reliability and Diagnostic Performance

The indexes showed a moderate or good inter-observer reliability. The overall inter-observer ICC (Intra-class correlation coefficient) was 0.524 (CI 95%: 0.304–0.738) for TDI; 0.793 (CI 95%: 0.646–0.9) for ACI; 0.504 (CI 95%: 0.283–0.724) for PCI.

The indexes had a moderate accuracy. The measurement performed observing radiographic images showed a mean % error with respect to the gold standard represented by the open measurements (Δ accuracy) of − 18.9% (CI 95%: − 28.5% to − 9.4%) for TDI; + 13.5% (CI 95%: 2.3–24.6%) for ACI; + 5.3% (CI 95%: 0.2–10.3%) for PCI.

**Fig. 4:**
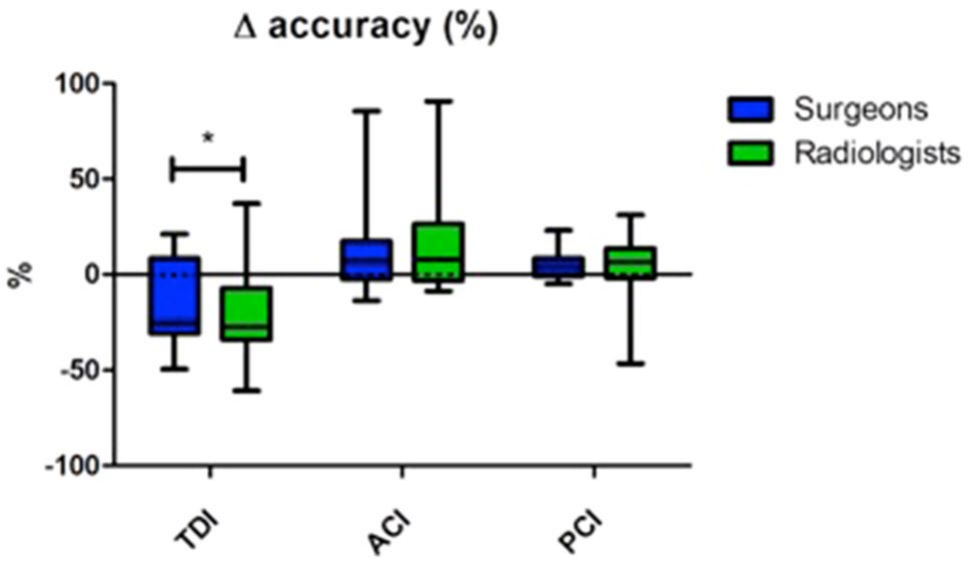
Box-and-whiskers plots illustrating the comparison between the accuracy observed for the subgroups of orthopaedic surgeons and musculoskeletal radiologists for the three indices (*p value < 0.05). The solid line indicates the perfect accuracy (difference = 0 with respect to the real value)

The Δ accuracy (where Δ equal to 0 represents the perfect accuracy) reported in the subgroup of orthopaedic surgeons was -15.5% (CI −24.8% to −6.2%) for TDI, + 12.3% (CI 95 2.1–22.4%) for ACI and + 4.9% (CI 95 1.4–8.4%) for PCI. The accuracy reported in the subgroup of musculoskeletal radiologists was −22.5% (CI − 32.0% to − 13.0%) for TDI, + 14.7% (CI 95 3.8–25.6%) for ACI and + 5.6% (CI 95 − 1.4–12.7%) for PCI. Only for the TDI a statistically significant difference in accuracy between orthopaedic surgeons and musculoskeletal radiologists was observed (Fig. 4).

The precision was calculated as the mean error of the measurements on digital radiographs with respect to open measurements, and it resulted ± 12% (95% CI 7.7–16.3%) for TDI, ± 6% (95% CI 3.5–8.5%) for ACI and ± 6% (95% CI 1.0–11%) for PCI.

### Responsiveness: Comparison Between Native Elbows and Simulated Coronoid Fracutre

The mean radiographic TDI was 0.45 ± 0.03 (range 0.40–0.51) in native elbows and 0.39 ± 0.08 (range 0.29–0.51) after simulated coronoid fracture (*p* = 0.035). The mean open TDI was 0.60 ± 0.09 (range 0.48–0.73) in native elbows and 0.48 ± 0.14 (range 0.31–0.73) after simulated coronoid fracture (*p* = 0.02) (Fig. 1C).

The mean radiographic ACI was 1.90 ± 0.17 (range 1.66–2.22) in native elbows and it resulted 1.58 ± 0.21 (range 1.22–1.89) after simulated coronoid fracture (p = 0.001). The mean open ACI was 1.76 ± 0.25 (range 1.14–1.88) in native elbows and 1.39 ± 0.27 (range 0.90–1.88) after simulated coronoid fracture (*p* = 0.001) (Fig. 2C).

The mean radiographic PCI was 1.36 ± 0.08 (range 1.24–1.48) in native elbows and 1.32 ± 0.16 (range 0.92–1.48) after simulated coronoid fracture (*p* = 0.387). The mean open PCI was 1.28 ± 0.14 (range 1.05–1.56) in native elbows and 1.28 ± 0.14 (range 1.05–1.56) after simulated coronoid fracture (*p* = 1). As expected, no significant changes between native and fractured specimens were observed in this index measuring posterior coverage.

The effect size (Cohen’s *d*) based on the open measurements (gold standard) was 0.981 for TDI and 1.394 for ACI. For what concern the effect size based on the radiographic measurements, it resulted 1.057 for TDI and 1.684 for ACI. These values correspond to large or very large effect sizes [31], suggesting that these indexes are effective in the identification of differences between native and fractured elbows. Since PCI did not vary between fractured and native elbows (effect size = 0) it was not considered for the linear model development.

### Predictive Potential: ACI and TCI as Indicators of Fracture

Since ACI and TDI demonstrated to significantly change in fractured elbows compared to native elbows, these two parameters were considered as possible indicators of fractures. The variable “fracture” was set as a dependent categorical variable (fractured = 1; native = 0) in a generalized linear model using radiographic ACI and TDI values (mean of all observers) as independent variables (or “predictors”). The multiple regression analysis showed that only ACI was able to significantly influence the result (*p* < 0.05); nevertheless, TDI was maintained in the model even if not significant since it allowed for an improvement of model fitting. The model equation (18.473-ACI*9.289-TDI*5.409) was able to identify a fracture with a sensitivity of 80% and a specificity of 100%. The area under the relative ROC curve (AUC) was 0.88, confirming the good performance of the test.

The application of the model test using the open measurements (study gold standard) resulted in a 90% sensitivity and 90% specificity.

## Discussion

The main finding of this study is that all the proposed radiological indexes demonstrated a good–moderate inter-observer reliability, accuracy and precision, well reproducing the open measurements considered as gold standard. ACI was the most reliable parameter to be used in the discrimination of native and fractured elbows, and the use of radiographs for its determination was reliable.

Since the calculation of the indexes of interest is determined by measurement obtained from radiographic images, the quality of these measure represents an important step in the validation of their use.

Interclass correlation among observers resulted moderate for TDI and PCI (> 0.5) and good for ACI (> 0.7), confirming the reliability of these measures, in particular for ACI.

ACI also demonstrated good mean precision (± 6%) and better accuracy compared to TDI (+ 13.5% vs. − 18.9%). PCI also demonstrated good precision (± 6%) and the best accuracy among the evaluated indexes (+ 5.3%) but, as expected, it was also the only index not changing significantly between native and fractured elbows. On the contrary, TDI and ACI decreased significantly after simulated coronoid fracture, with this result being confirmed both by gold standard open measurements and by radiographic measurements, supporting the effectiveness of these evaluations in the identification of fractures. Large and very large effect sizes were observed for TDI and ACI, respectively, when comparing native and fractured elbow with both radiographic and open measurements, suggesting that these indexes are effective in the identification of fractures.

The moderate interclass correlation reported for TDI and PCI may be due to the difficulty of obtaining an adequate standard plane in lateral view X-rays, and a consequent difficulty in identifying the reference points. The use of 3D-images (CT, MRI) could solve this problem in a trauma setting.

Elbow fracture diagnosis can be challenging; detection rate has been reported to be different between orthopaedic surgeons and radiologists, attesting the elbow as the most overlooked site among the upper limb [34, 35]. Standard elbow views are not always enough to avoid missed diagnoses [36, 37]. In a recent X-ray and CT comparison study, 12% of patients with positive extension test and normal radiography had an occult fracture [38].

Even though X-rays are suitable to be interpreted by a wide range of clinicians, objective and reliable indexes are mandatory to better assess the functional morphology of the elbow and to raise suspicion of coronoid fracture. Furthermore, such indexes of congruency between the GSN and the distal humerus could be helpful in defining elbow morphologies at risk of acute and chronic instability, as well as to plan tailored treatment of elbow stiffness. Measurements of olecranon and coronoid height and GSN congruency on plain radiographs have already been proposed decades ago, yet as isolated reports and without undergoing a strict a validation process [39]. Subsequent studies focussed mainly on the role of the coronoid process, with the anatomy of the GSN as a whole structure fading to the background, until a recent MRI-based investigation by Giannicola et al. defined the normal values of the “ulnar greater sigmoid notch coverage angle” as a parameter to evaluate the GSN congruency [40].

The linear measurements collected in our study permit to evaluate the GSN anatomy with the angle RCA, which can be derived by a mathematical operation [*RCA* = *4 arctan (2 BD/AC)*]. Being linear measurements simpler and more reproducible than angular measurements, we discourage a direct angular measurement of the GSN, provided the aforementioned reference points are well identifiable on standardized plain lateral radiographs. Herewith, the ACI, the PCI and the TDI could be implemented in the radiographic workflow of posttraumatic elbow and in the evaluation of joint instability risk factors involving the coronoid process. These indexes correlate with the height of the coronoid process and inversely with the depth of the GSN; in this way, they completely define the containing capacity of the proximal ulna. In case of instability, these indexes could be associated with other, already described, radiological signs such as the “vacuum sign” visible on stress radiographies [41].

Further studies will define how the new elbow radiographic indexes proposed in this study perform in describing the functional elbow anatomy, allowing early recognition of patients with elbow instability risk factors in clinical practice.

These indexes could also play a key role in degenerative elbow pathology; in this context, they could simplify the surgical decision-making by identifying those patients with anatomical risk factor for developing stiffness.

In the search for a test able to identify fractures, a formula obtained combining the values of ACI and TDI indexes in a generalized linear model demonstrated good performances, identifying fractures with a sensitivity of 80% and a specificity of 100%. Despite the good performance of the test, it suffers of several limitations. The use of the same measurements for model development and test assessment represents the main bias. In addition, the low number of cases, as well as the lack of significance in the TDI parameter of the model, does not allow for the generalization of the test application.

Equivalent radiographic indexes have been used to evaluate the continence of concave joints in other anatomical districts [42–45].

Currently, no radiological index is commonly used in the evaluation of intrinsic stability, except for dynamic ultrasound stress tests [46]. Though, plain films are still unable to guarantee the benefits of dynamic investigation, several dynamic stress while performing X-rays were performed without clinically relevant results [47, 48]. Thank to the rigorous ex vivo anatomical validation, this study paves the way to the application of the described indexes and angles in vivo (*Part B*, [49]) and in the clinical setting, anticipating also a possible future description on CT and MRI images reconstructed in a sagittal trochlear plane.

Several limitations should be considered for this study. First, this is a no blinded study with a small sample size, which could amplify bias related to technical procedural aspects and anatomical variants. Furthermore, the contribution of muscle tone to elbow stability could not be investigated in an ex vivo study [50]. To minimize bias, care was taken in evaluating the specimens for visible signs of gross instability, deformity and previous trauma and specimen moisture and temperature were maintained at constant levels throughout the whole study.

Despite these limitations, this is the first study that used the anatomical measurements as gold standard for the validation of a radiographic study of elbow functional anatomy. These indices are mainly making it easy to diagnose a coronoid fracture. However, elbow instability depends on a complex interplay between soft tissues and bony structures, of which the coronoid fracture plays a key role. The inclusion of the angles described in the manuscript may add more value to the regression equation or calculations. Future studies are needed to clarify the clinical implications of these indexes.

## Conclusion

Three new, simple and easily reproducible radiological indexes to describe the congruency of the greater sigmoid notch have been proposed. TDI and ACI change significantly after a simulated coronoid fracture, indicating a good responsiveness of these parameters to a pathological condition. Furthermore, combining TDI and ACI in a regression model equation allowed to identify simulated fractures with high sensitivity and specificity.

The newly proposed indexes are, therefore, promising tools to improve diagnostic accuracy of coronoid fractures and show potential to enhance perioperative diagnostic also in cases of elbow instability and stiffness.

## Abbreviations

GSN: Greater sigmoid notch
TDI: Trochlear Depth Index
PCI: Posterior Coverage Index
ACI: Anterior Coverage Index
RCA: Radiographic coverage angle
ODA: Olecranon–diaphisary angle
SD: Standard deviation
ICC: Intra-class correlation coefficient

## Declarations

### Study Guarantor

Francesco Luceri, M.D.
